# Supplementary material for: Association of intrauterine synechiae with pituitary gonadotrophin pulse patterns: A pilot study
Source: PLoS One. 2023 Dec 15;18(12):e0289075. doi: 10.1371/journal.pone.0289075 (PMC10723672; doi:10.1371/journal.pone.0289075)
Supplement: Attachment — Submitted filename: Response to reviewers.docx [file pone.0289075.s003.docx]

São Paulo, April 5, 2023

**Re: Submission of manuscript entitled “Association of refractory Asherman syndrome with pituitary gonadotrophin pulse patterns: A pilot study”**

Dear Prof Editor,

We would like to resubmit our manuscript entitled “Association of Refractory Asherman syndrome with pituitary gonadotrophin pulse patterns: A pilot study,” after corrections.

**Reviewer #1**

1. **In abstract sector: you mentioned this phrase (In both groups, blood was collected during the follicular phase of the menstrual cycle). You should revise and readjust to avoid misleading readers, as group one presented with amenorrhea.**

**A:** The serial ultrasound was performed to evaluate the cycle. We included in abstract.

2. **In abstract sector: you mentioned (During the first hour of monitoring, cumulative FSH pulsatile frequency was lower in the AS group than in the control group within the next 3 h of measurements). Rewrite the phrase more clearly**

**A:** We rewrote it

3. **In introduction section, defining Asherman syndrome has about 11 references, what is the rationale for this great number? Can we minimize especially no benefit from the repetition?**

**A:** We reduced the references.

4. **-The study was retrospective: In methodology section, the statement (These patients had amenorrhea for at least six months after admission to the study). May be understood. You mean before admission to the study? Also, I just want to confirm that 8 patients refused to participate?**

**A:** We clarified on the manuscript. Eight patients refused to collect blood.

5. **In methodology section, Clarify the meaning of (The same was done for women with Asherman syndrome, exception the analysis of gonadotropins and sex steroids, estrogen, and progesterone.).**

**A:** The formula used to determine the mean time of occurrence of pulses.

6. **You have a reference for this blood sampling protocol? (For the collection of blood samples, the following protocols were followed: a) patients were fasted and remained in the horizontal supine position; b) intravenous puncture was performed with a size-19 catheter, and the vein was maintenance with a saline solution drip (20 drops per minute); and c) 25 blood samples were collected from each woman**)

**A:** This protocol was used at the time this study was done.

7. **In the phrase (We observed that there was no statistically significant difference between the mean values of serum concentrations of LH, FSH, and progesterone, or in the mean serum concentration levels of estradiol (p>0.05) The (p value) equal or more? confirm.**

**A:** P critical values was 0,05. Then, P-values accepted as statistically significant are lower than 0,05.

8. **In discussion section: what you mean by (The influence of hormonal status at the time of injury and relevant factors on genesis). There are missing words? I don’t understand.**

**A:** There was Mistyping in this phrase, instead of “and” we will use the word “are”.

9. **I discussion section and at the end of page 10 in (pdf copy) *(They observed the occurrence of a pulse every 100-120 minutes in the follicular phase of normal women).* The word they refer to whom???**

**A:** We rewrite this phrase, the word “they” is referring to the researchers (Reame *et. al*.)

10. **You abbreviated Asherman to AS in introduction section, however, I noticed you mention it again with full . Revise that allover the article.**

**A:** We revised and changed the term “Asherman syndrome” and replaced with “Intrauterine Synechiae” (IS) using only the abbreviation after the first use of the full term.

**Reviewer #2**

1. **Why did you choose the term “Asherman syndrome”. It is well known that Asherman syndrome is intrauterine synechia following surgical evacuation. The term “intrauterine synechia” is a more accepted term.**

**A:** We agree, Intrauterine Synechiae is a more accepted term, every time we used Asherman Syndrome we will change it for Intrauterine Synechiae

2. **In the introduction section, in the last paragraph “correct “his” to “this”.**

**A:** The correct word is “this”. Rewrote

3. **You collected 25 samples from each patient, and this was approved by your IRB. What was your rational for this? I mean what is the benefit that the patient was told to expect? I would be grateful if you would share your IRB. Additionally maintaining the IV line with saline infusion, would this not alter your results? Please explain.**

**A:** At the time the research was carried out there was a protocol for investigating infertility In patients with intrauterine synechiae, involving also investigating possible Hypothalamic-pituitary-ovarian axis disorder through the gonadotrophins pulsatility study, during the first four hours every ten minutes.

The benefit to patients was to detect possible disorders in the Hypothalamic-pituitary-ovarian axis.

Saline solution dripping aimed to maintain venous permeability in a minimum quantity in both groups indistinctly. We wrote a phrase explaining how this may be a limitation of our study.

IRB number is: IRB# 4225739-CAAE 20637019400000068

4. **What is meant by “The G1 patients were followed by serial ultrasounds, and the ultrasound was performed in the next cycle when there were at least five small cystic images in one or both ovaries, which could suggest an early follicular phase”**

**A:** As most of G1 patients presented amenorrhea, serial ultrasound could suggest an early follicular phase, so we can schedule sample collection in these patients.

5. **The discussion is well written but please express your points of strength and limitation.**

**A:** As strength we consider, the association of Hypothalamic-pituitary-ovarian axis disorder to the presence of intrauterine synechiae through the observations of gonadotrophins pulsatility. As limitations we considered, small sample size.

**Reviewer #3**

1. **Although the concept is interesting and Asherman syndrome is not frequent BUT this does not justify the very low number of participants in this study. Asherman syndrome has classification of severity: it seems that the authors included only the severe cases with Amenorrhea: pls explain why not cases with moderate IU adhesions.**

**A:** patients were women with severe intrauterine adherences, at the time these patients were followed at outpatient facilities and accepted to participate. We did not choose these patients nor excluded moderate IU adhesions.

2. **The following statement in the manuscript is very unusual in research design: "These patients had amenorrhea for at least six months after admission to the study." why to wait six month after recruitment !!!!!!**

**A:** We did not wait 6 months, when these women were recruited, they already presented 6 months or more amenorrhea.

3. **The authors failed to mention which day of cycle blood samples were taken: is it the same day for all the participants for example cycle day 11.**

**A:** Its not the same day for all participants, it was almost impossible to determine the cycle day, especially for those with amenorrhea. In these patients we use ultrasound suggesting that the patients were in early follicular phase.

4. **The authors failed to correlate the level of hormones with the follicle size at the day of sampling: what if day of sampling was when follicle 11mm in Asherman cases and follicle 15mm in control group: Hormonal assays differ according to follicular activity**

**A:** Actually, this situation can be considered as a limitation of our study.

Additional comments to be clarified

5. **Exclusion criteria what about women with endocrinological disorders especially PCOS women**

**A:** Yes, we agree, PCOS should have been used as an exclusion criterion.

6. **How was the diagnosis of refractory AS established based on what criteria**

**A:** Patients has intrauterine adhesions confirmed by hysterosalpingography and hysteroscopy

7. **Was the trial registered and what is the registration number
   How was the study retrospective**

**A:** The study was not registered as a trial. O IRB Number IRB# 4225739-CAAE 20637019400000068.

The study design is not retrospective. The study was done prospectively from group definition and baseline measurements to 4 hours follow up. The study was done in 1993 and we reevaluated the data.

8. **Clarify the duration of fasting before sampling**

**A:** 8-12 hours

9. **Please discuss the effect of saline infused on the results of sampled blood especially its dilutional effect.**

**A:** Saline solution dripping aimed to maintain vein permeability, it possible that this situation may interfere in results affecting both groups.

10. **More details about kits and its sensitivity**

**A:** We used LH Fluorometric Enzyme immunoassay and FSH Fluorometric Enzyme immunoassay and FSH, Baxter Diagnostics Inc.

We did not review its sensitivity.

11. **Define primary and secondary outcomes with references
    Remove amenorrhea duration and menstrual cycle from table 1
    In discussion.**

**A:** Our primary outcome was gonadotrophins pulsatility and it was defined by Root et. al. (reference number 24). Hormone levels related to IS were used as an outcome by several authors, take as example our bibliographic references: 1-4, 17-19, 20-23.

We removed all amenorrhea from table 1.

12. **Physiological explanation of findings, strengths and limitations of the study should be added to discussion. Master data lack age.**

A: Intrauterine synechiae development may promote a decrease in activity of uterine muscular wall, reducing the perfusion of sexual hormones at endometrial level contributing to endometrium atrophy.

We added to discussion a paragraph about limitations ands strengths of our study. Age is in database.
