# Supplementary material for: Association of intrauterine synechiae with pituitary gonadotrophin pulse patterns: A pilot study
Source: PLoS One. 2023 Dec 15;18(12):e0289075. doi: 10.1371/journal.pone.0289075 (PMC10723672; doi:10.1371/journal.pone.0289075)
Supplement: Attachment — Submitted filename: Response to reviewers 2.docx [file pone.0289075.s004.docx]

Sao Paulo, July 5, 2023

**Dear Plos One editors and reviewers**

We would like to submit our manuscript entitled **“Association of intrauterine synechiae with pituitary gonadotrophin pulse patterns: A pilot study”**. We already rewrote several term as suggested by Reviewer # 1. Now we send you this corrected version including the Reviewer#2 suggestions. All authors agree with this version, we hope to meet the required changes and to clarify your questions about our work.

1- In abstract sector: you mentioned this phrase (In both groups, blood was collected during the follicular phase of the menstrual cycle). You should revise and readjust to avoid misleading readers, as group one presented with amenorrhea.

A1: Blood sample were collected every 10 minutes during a 4-h period. The serial ultrasound was performed in both groups for evaluating the cycle phase. Blood was collected when the follicles size was between 5-10 mm.

2- In abstract sector: you mentioned (During the first hour of monitoring, cumulative FSH pulsatile frequency was lower in the AS group than in the control group within the next 3 h of measurements). Rewrite the phrase more clearly

A2: We changed the phrase to: “During the first hour of monitoring, cumulative FSH pulsatile frequency was lower in the IS group than in the control group”.

3- In introduction section, defining Asherman syndrome has about 11 references, what is the rationale for this great number? Can we minimize especially no benefit from the repetition?

A3: We checked and removed 5 references.

4- The study was retrospective:

- In methodology section, the statement (These patients had amenorrhea for at least six months after admission to the study). May be understood. You mean before admission to the study?
- Also, I just want to confirm that 8 patients refused to participate?

A4: We mean “before admission”, we rewrote the phrase:

“Uterine synechiae was present in the cases group (G1) experiencing amenorrhea for at least six months before the study admission”.

Yes, 6 patients in the cases group (G1) and 2 in the control group (G2) refuse to participate in the study, refused to participate when evaluated in the outpatient endocrine gynecologic clinic.

5- In methodology section, Clarify the meaning of (The same was done for women with Asherman syndrome, exception the analysis of gonadotropins and sex steroids, estrogen, and progesterone.).

A5: We wrote incorrectly the phrase: we meant “the same exclusion criteria were used for the cases group (G1)”

(Page 4)

6- You have a reference for this blood sampling protocol?

The protocol was done following the methods used in reference number 22:

- Fenichel RM, Dominguez JE, Mayer L, Walsh BT, Boozer C, Warren MP. Leptin levels and luteinizing hormone pulsatility in normal cycling women and their relationship to daily changes in metabolic rate. Fertil Steril. 2008 Oct;90(4):1161-8

7- In the phrase (We observed that there was no statistically significant difference between the mean values of serum concentrations of LH, FSH, and progesterone, or in the mean serum concentration levels of estradiol (p>0.05)).

The (p value) equal or more? confirm.

A7: The p value used to define statistical differences was less than 0.05, we already fix this typing error (Page 7)

8- In discussion section: what you mean by (The influence of hormonal status at the time of injury and relevant factors on genesis). There are missing words? I don’t understand.

We replaced with: “The influence of hormonal status at the time of injury are relevant factors on genesis (28)”

(Page 7)

9- I discussion section and at the end of page 10 in (pdf copy) *(They observed the occurrence of a pulse every 100-120 minutes in the follicular phase of normal women)*

The word they refer to whom???

A9: We are referring to the researchers of the cited study.

We rewrote: “The researchers observed the occurrence of a pulse every 100-120 minutes in the follicular phase of normal women”

(page 8)

10- You abbreviated Asherman to AS in introduction section, however, I noticed you mention it again with full. Revise that allover the article.

A10: We already replaced the term Asherman syndrome to Intrauterine synechiae and used the abbreviation “IS”.
