# Supplementary material for: Association of intrauterine synechiae with pituitary gonadotrophin pulse patterns: A pilot study
Source: PLoS One. 2023 Dec 15;18(12):e0289075. doi: 10.1371/journal.pone.0289075 (PMC10723672; doi:10.1371/journal.pone.0289075)
Supplement: Attachment — Submitted filename: Reviewer comments.docx [file pone.0289075.s002.docx]

Thank you for the invitation for reviewing this interesting study , aiming for discussing association of refractory Asherman syndrome with pituitary gonadotrophin pulse Patterns and recommended that a specific clinical protocol may be necessary for estrogen treatment of these patients. I'm asking the authors for clarification of the next items.

1- In abstract sector: you mentioned this phrase (In both groups, blood was collected during the follicular phase of the menstrual cycle). You should revise and readjust to avoid misleading readers, as group one presented with amenorrhea.
2- In abstract sector: you mentioned (During the first hour of monitoring, cumulative FSH pulsatile frequency was lower in the AS group than in the control group within the next 3 h of measurements). Rewrite the phrase more clearly
3- In introduction section, defining Asherman syndrome has about 11 references, what is the rationale for this great number? Can we minimize especially no benefit from the repetition ?.
4- The study was retrospective :

- In methodology section, the statement (These patients had amenorrhea for at least six months after admission to the study). May be understood. You mean before admission to the study?
- Also, I just want to confirm that 8 patients refused to participate?

5- In methodology section, Clarify the meaning of (The same was done for women with Asherman syndrome, exception the analysis of gonadotropins and sex steroids, estrogen, and progesterone.).
6- You have a reference for this blood sampling protocol ?

(For the collection of blood samples, the following protocols were followed: a) patients were fasted and remained in the horizontal supine position; b) intravenous puncture was performed with a size-19 catheter, and the vein was maintenance with a saline solution drip (20 drops per minute); and c) 25 blood samples were collected from each woman)

7- In the phrase (We observed that there was no statistically significant difference between the mean values of serum concentrations of LH, FSH, and progesterone, or in the mean serum concentration levels of estradiol (p>0.05)).

The (p value) equal or more? confirm.

8- In discussion section: what you mean by (The influence of hormonal status at the time of injury and relevant factors on genesis). There are missing words? I don’t understand.
9- I discussion section and at the end of page 10 in (pdf copy) *(They observed the occurrence of a pulse every 100-120 minutes in the follicular phase of normal women)*

The word they refer to whom???

10- You abbreviated asherman to AS in introduction section , however , I noticed you mention it again with full . Revise that allover the article .
